# Field and greenhouse application of an attract‐and‐kill formulation based on the yeast *Hanseniaspora uvarum* and the insecticide spinosad to control *Drosophila suzukii* in grapes

**DOI:** 10.1002/ps.6748

**Published:** 2021-12-15

**Authors:** Urban Spitaler, Carlo S Cossu, Lorenz Delle Donne, Flavia Bianchi, Guillermo Rehermann, Daniela Eisenstecken, Irene Castellan, Claire Duménil, Sergio Angeli, Peter Robatscher, Paul G Becher, Elisabeth H Koschier, Silvia Schmidt

**Affiliations:** ^1^ Entomology Group, Institute for Plant Health, Laimburg Research Centre South Tyrol Italy; ^2^ Institute of Plant Protection, Department of Crop Sciences University of Natural Resources and Life Sciences Vienna Austria; ^3^ Laboratory for Flavours and Metabolites, Institute for Agricultural Chemistry and Food Quality Laimburg Research Centre South Tyrol Italy; ^4^ Chemical Ecology – Horticulture, Department of Plant Protection Biology Swedish University of Agricultural Sciences Alnarp Sweden; ^5^ Faculty of Science and Technology Free University of Bozen‐Bolzano South Tyrol Italy

**Keywords:** grapes, integrated pest management, invasive fruit pest, precision agriculture, spotted wing drosophila

## Abstract

**BACKGROUND:**

The invasive insect *Drosophila suzukii* (Matsumura) is an important pest of several red grape varieties. The yeast *Hanseniaspora uvarum* (Niehaus), which is associated with *D. suzukii*, strongly attracts flies and stimulates them to feed on yeast‐laden food. In the present study, a formulation based on *H. uvarum* culture with spinosad insecticide was applied to the foliage of vineyards and control of *D. suzukii* was compared to applying spinosad to the whole plant. After successful *H. uvarum* and insecticide application in the vineyard, we tested additional *H. uvarum*‐based formulations with spinosad in a greenhouse to determine their capacity to control *D. suzukii*.

**RESULTS:**

Application of the *H. uvarum*‐spinosad formulation at 36.4 g of spinosad per hectare reduced the *D. suzukii* field infestation at the same rate as applying 120 g of spinosad per hectare and prevented spinosad residues on grapes. Leaves treated with *H. uvarum* and spinosad in the field and transferred to a laboratory assay caused high mortality to flies and reduced the number of eggs laid on fruits. Formulations with spinosad applied in the greenhouse showed that both *H. uvarum* culture and the yeast cell‐free supernatant of a centrifuged culture increased fly mortality and reduced the number of eggs laid compared to the unsprayed control.

**CONCLUSION:**

In comparison to typical spinosad spray applications, the use of *H. uvarum* in combination with spinosad as an attract‐and‐kill formulation against *D. suzukii* reduces pesticide residues on the fruits by targeting the treatment to the canopy and decreasing the amount of insecticide per hectare without compromising control efficacy.

## Graphical abstract

Application of the yeast *Hanseniaspora uvarum* in a control strategy against *Drosophila suzukii* reduces insecticide residues on grapes by targeting the canopy, which does not compromise the control efficacy.

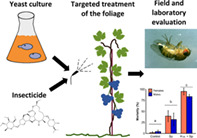

© 2021 The Authors. *Pest Management Science* published by John Wiley & Sons Ltd on behalf of Society of Chemical Industry.

## 1 INTRODUCTION

*Drosophila suzukii* (Matsumura) (Diptera: Drosophilidae), also known as spotted wing drosophila, is an important insect pest of soft‐ and thin‐skinned fruit crops, including berries, stone fruit and grapes.^1^ The control of *D. suzukii* usually relies on the application of insecticides to the whole plant to reduce yield losses.^2^ Unfortunately, most insecticides result in fruit residues and are not selective.^3^ New strategies based on insect semiochemicals could reduce the amount of insecticide applied in the field and prevent residues that remain on the fruit.^3^, ^4^ Combining insecticide with an attractant that guides the flies to the insect toxic bait might allow for the targeted application to the canopy while avoiding the fruit.^4^, ^5^ Such a strategy could promote more sustainable and targeted chemical control. Successful attempts to develop an attract‐and‐kill strategy against *D. suzukii* were previously conducted with formulations based on *Saccharomyces cerevisiae* and *Aureobasidium pullulans* in cherry orchards and with a complex formulation of unknown ingredients in combination with conventional treatments in blueberry and raspberry fields.^6^, ^7^

For *D. suzukii*, yeasts are considered suitable lures for attract‐and‐kill control strategies since they act as feeding stimulants^8^, ^9^ and are an important source of nutrients for this pest.^4^, ^10^, ^11^ One of the most relevant yeasts is *Hanseniaspora uvarum* (Niehaus), which was found in *D. suzukii*‐infested grapes and raspberry fruits,^12^, ^13^ as well as in *D. suzukii* adults and larvae.^6^, ^11^, ^14^ The yeast *H. uvarum* is more attractive and phagostimulatory toward *D. suzukii* than other investigated yeast species.^9^, ^10^, ^15^ Furthermore, *H. uvarum* is naturally present on grapes, therefore its presence likely would not interfere with winemaking.^16^, ^17^ Control methods based on *H. uvarum* and insecticides were previously tested in the laboratory and greenhouse, and led to reduced oviposition and higher mortality of *D. suzukii* adults.^5^, ^6^, ^8^, ^18^, ^19^

Attraction to yeast is strain‐specific,^20^ therefore an *H. uvarum* strain that has been extensively studied and is attractive to *D. suzukii* was used in the present study. The *H. uvarum* strain LB‐NB‐2.2 was isolated from feeding galleries of *D. suzukii* larvae in infested grape berries of the variety Vernatsch in South Tyrol in 2012.^12^ This strain was previously shown to act as a feeding stimulant and attractant for *D. suzukii* females, and it was successfully used as an attractive component in control strategies in greenhouse assays.^5^, ^10^, ^19^ Furthermore, the intra‐ and extracellular concentrations of compounds such as amino acids, carbohydrates, sugar alcohols, organic acids and lipids for the culturing of this *H. uvarum* strain grown in liquid medium were previously characterized,^10^, ^21^ and the persistence of nutritional and volatile compounds on the surface of grape leaves of potted plants treated with an attract‐and‐kill formulation based on this *H. uvarum* strain was described.^5^

Among the numerous insecticides that can be used against *D. suzukii*,^22^, ^23^, ^24^, ^25^, ^26^ some have been tested in combination with *H. uvarum*.^6^, ^8^, ^19^ Spinosad, which can be used in integrated and organic production,^22^ was proven to be effective against *D. suzukii* based on laboratory and greenhouse trials.^5^, ^18^ Therefore, this insecticide was chosen in combination with the yeast *H. uvarum* LB‐NB‐2.2 for our study.

The soft‐skinned red grape variety Vernatsch (alternative names Schiava in Italy, Trollinger in Austria and Germany), which is used for winemaking, has a lower penetration resistance against *D. suzukii* oviposition than other grape cultivars.^27^, ^28^ The dispersal of *D. suzukii* has compromised the cultivation of Vernatsch since the first appearance of this pest in 2009.^2^ Great damage due to *D. suzukii* infestation occurs, especially when the penetration resistance of the berry decreases before harvest and when temperatures are mild and precipitation occurs.^29^, ^30^

Our objective was to determine whether the combined application of *H. uvarum* and spinosad in vineyards could restrict the spray application to the foliage and reduce areal insecticide release and residues on grapes without compromising the control efficacy relative to conventional treatment of the whole plant. In the laboratory, *D. suzukii* flies were exposed to leaves collected in the field after treatment to obtain additional information about its effect in the vineyard. Furthermore, this study explores the effect of different *H. uvarum* formulations that can be used for storage of yeasts to develop sustainable and cost‐effective attract‐and‐kill strategies. Residual analyses were performed to better understand the persistence of the applied insecticide.

## 2 MATERIALS AND METHODS

### 2.1 Yeast cultures and formulations

All assays were performed with the yeast *H. uvarum* (strain LB‐NB‐2.2, accession number GenBank NCBI: MK567898). This *H. uvarum* strain was isolated in 2012 from *D. suzukii*‐infested grapes.^12^

The first *H. uvarum* culture was industrially manufactured by Agrifutur srl (Alfianello, Italy) in a 40‐L fermenter under aerobic conditions on potato dextrose broth (4 g L^–1^ peptone from potato, 20 g L^–1^ dextrose) at 25 °C for 30 h, and it had a pH value of 4.1 and a cell density of 4.8 × 10^7^ cells per mL. The second *H. uvarum* culture was cultivated in the laboratory at the Laimburg Research Centre in 4 L of potato dextrose broth (24 g L^–1^; Difco, Becton–Dickinson, Le Pont de Claix, France) at 25 °C for 30 h in 6‐L Erlenmeyer flasks closed with cotton and aluminum foil on magnetic stirrers at 300 rpm, and it had a pH value of 4.0 and a cell density of 7.1 × 10^7^ cells per mL. For both cultures, media were inoculated with yeast cells grown on potato dextrose agar [4 g L^–1^ potato starch (from infusion), 20 g L^–1^ dextrose, 15 g L^–1^ agar; Difco, Becton Dickinson, Le Pont de Claix, France]. Both yeast cultures were used undiluted.

For the greenhouse assay, the industrially manufactured *H. uvarum* culture was preserved in three different formulations by Agrifutur srl. The formulations were *H. uvarum* culture without modifications before storage, *H. uvarum* supernatant obtained by centrifugation of the entire culture at 4000 rpm, and *H. uvarum* pellets obtained by centrifugation at 4000 rpm and subsequent freeze‐drying. All cultures or formulations were stored at −80 °C and thawed overnight at room temperature before use. The freeze‐dried *H. uvarum* pellets were diluted to the initial volume with distilled and autoclaved water after thawing.

### 2.2 Insects

A laboratory colony of *D. suzukii* in insect cages (BugDorm – 4M4590; MegaView Science Co., Ltd, Taichung, Taiwan) was maintained at 22 ± 1 °C, 65 ± 5% relative humidity, and 16 h photoperiod. The *D. suzukii* flies originated from infested fruits in South Tyrol, Italy and were reared on a *D. suzukii* cornmeal diet (previously designated DSCD(a) containing dry deactivated yeast) with living dry baker's yeast (RUF Lebensmittelwerk KG, Quakenbrück, Germany) sprinkled over the surface.^12^ The flies were also provided with a 5% sugar solution on cotton. Males and females that hatched together over 3 days were fed a cornmeal diet and sugar solution until the start of the experiment. When the flies reached an age of 5–8 days after emergence from the pupal stage, 20 females and 20 males were placed together in an insect cage (BugDorm – 1; MegaView Science Co., Ltd).

### 2.3 Vineyard trials

#### 2.3.1 Field application

**Figure 1:**
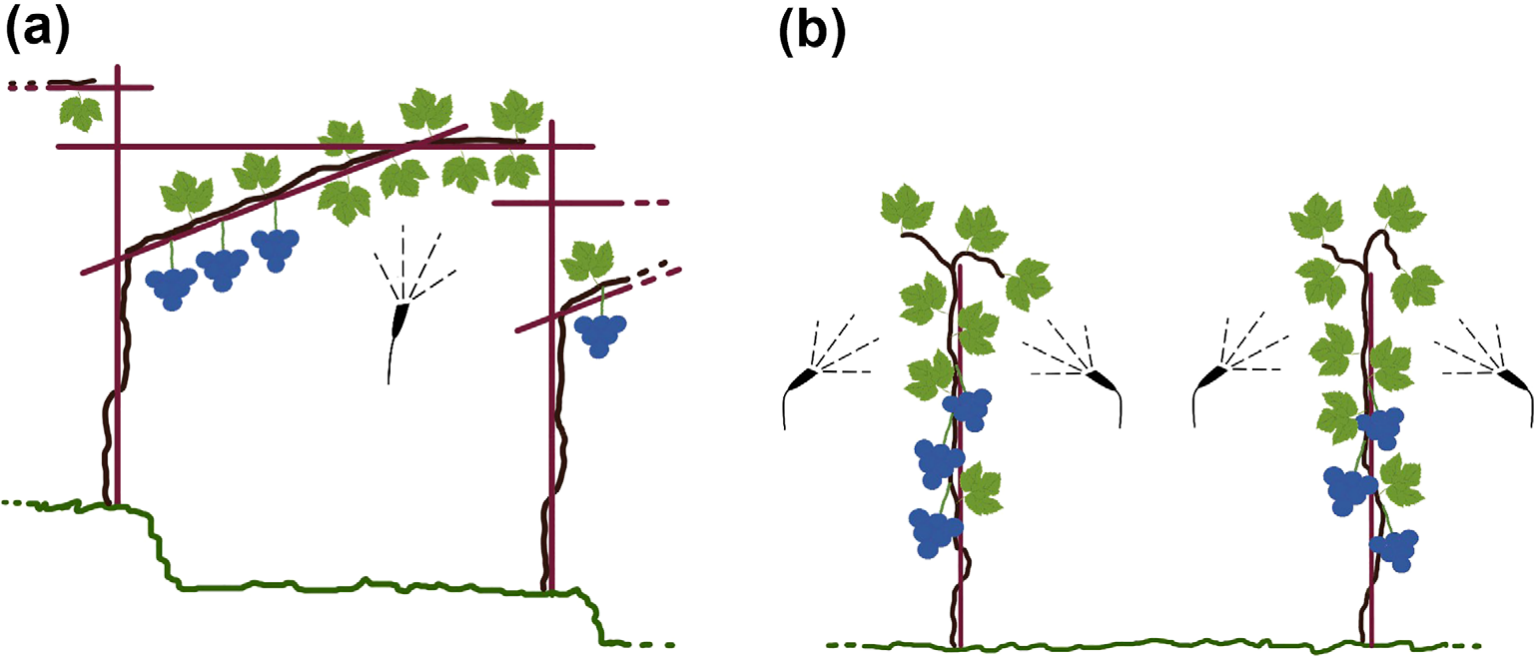
Illustration of the grapes trained (a) on a pergola and (b) with the Guyot method. The black spray patterns illustrate the targeted treatment of the foliage for both training types.

The field trials were performed in two vineyards that cultivate the local grape (*Vitis vinifera*) variety Vernatsch according to the guidelines for integrated fruit production in South Tyrol, Italy: at Schlossleiten (46°23′04.8” N, 11°17′10.6″ E), the grapes were cultivated using a pergola as the training method in 2019 and at Piglon (46°21′46.4” N, 11°17′21.0″ E), the grapes were cultivated with the single Guyot method in 2020 (Fig. 1). The experimental design consisted of three blocks, each containing one plot per treatment. The plots consisted of three rows and were 130 m^2^ in 2019 and 120 m^2^ in 2020. The plots were oriented adjacent to each other and perpendicular to the bordering edge of a forest, a *D. suzukii* infestation pressure point observed in previous years. The vineyard trials in 2019 and 2020 were performed between the end of August and the end of September.

The treatments were an unsprayed control, a conventional spinosad treatment applied to the whole plant and thawed *H. uvarum* culture + spinosad applied on the portion of the fruit‐free canopy. The *H. uvarum* culture applied in 2019 was industrially manufactured by Agrifutur srl, while the *H. uvarum* culture applied in 2020 was produced in the laboratory at the Laimburg Research Centre.

The conventional spinosad treatment contained 0.12 g spinosad (Laser, Dow AgroSciences Italia S.r.l., Milan, Italy; 480 g of spinosad per liter of product) per liter of water and was used at a spray rate of 1000 L of water per hectare. The applied amount resulted in 120 g of spinosad per hectare. The spinosad treatment was applied with a trailed airblast sprayer (AP 2/28 with axial fan; Lochmann GmbH, Italy) at 5.5 bar and 6 km h^–1^ through 12 black Albuz ATR 80° hollow cone nozzles (Agrotop Gmbh, Obertraubling, Germany) in 2019 and at 5.5 bar and 6.5 km h^–1^ through 12 red Albuz ADI 110° flat fan nozzles (Agrotop Gmbh) in 2020. On the pergola and on the Guyot system, one spinosad treatment was applied to both sides of each row.

The *H. uvarum* + spinosad treatment contained 0.1584 g of spinosad (0.33 mL of Laser) per liter of *H. uvarum* culture and was used at a spray rate of 230 L of yeast culture per hectare for both training systems. The applied amount resulted in 36.48 g of spinosad per hectare, which was added after thawing and was applied with an electric knapsack sprayer equipped with an anti‐drift fan nozzle CVI 110° green (Serena EL 16 LT; Italdifra Agricultural Tools S.r.l., Francofonte, Italy) at 2.5 bar. The manual application with the knapsack sprayer allowed a more precise treatment of the fruit‐free canopy. The amount of *H. uvarum* applied was previously tested to avoid dripping from the leaves to the ground. Targeted treatment of the fruit‐free canopy with *H. uvarum* + spinosad was possible for both training types (Fig. 1). On the pergola, one treatment was applied from below, and on the Guyot system, one treatment was applied on both sides of the plant. The treated area of the canopy had a width of approximately 80 cm.

**Figure 2:**
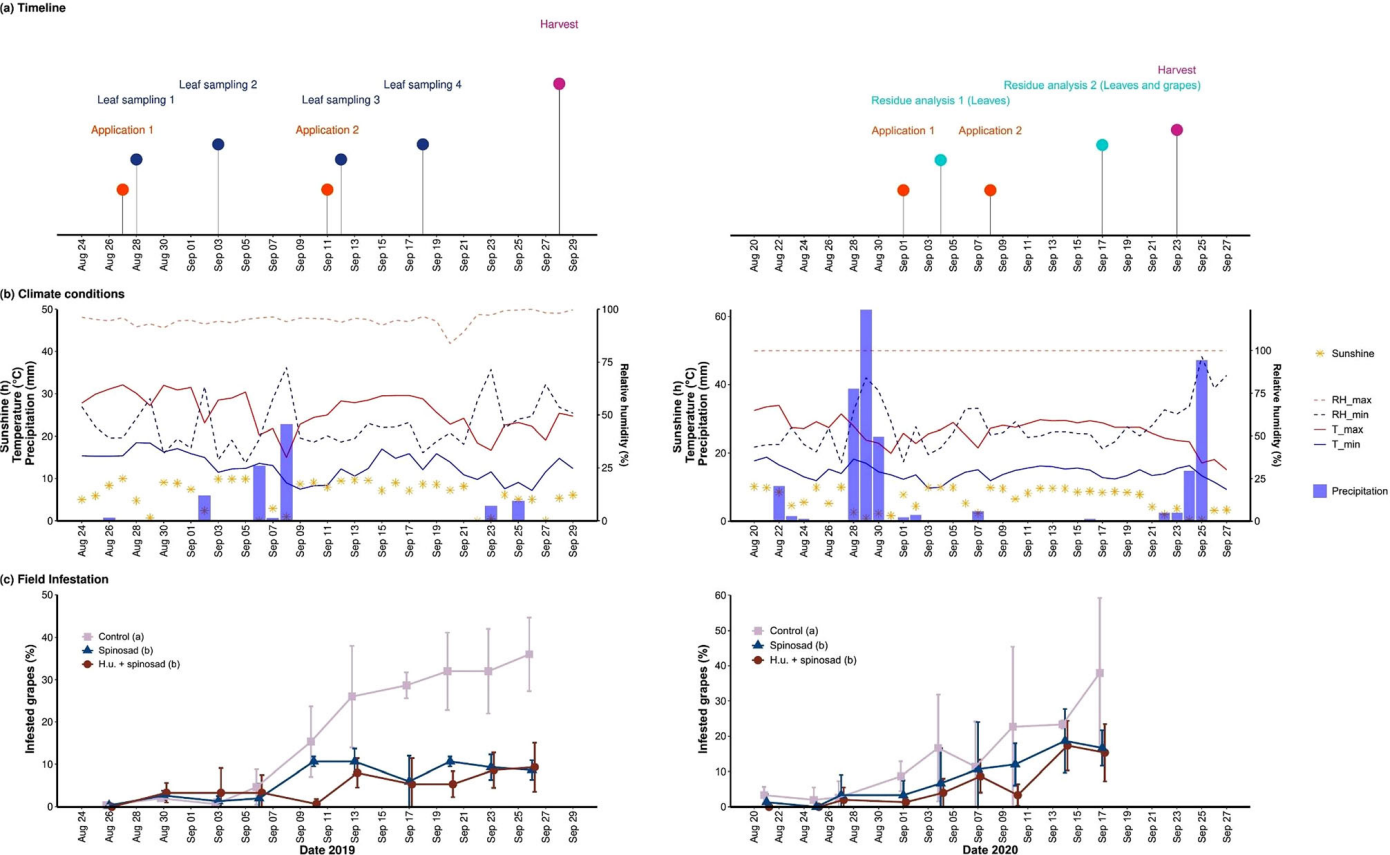
Effect of spinosad application with and without *H. uvarum* bait on *D. suzukii* field infestation of grapes trained with a pergola in 2019 (left) and with the Guyot method in 2020 (right). (a) Timeline with timepoints for the applications, leaf sampling for laboratory trials in 2019 (four samplings), leaf and grape sampling for spinosad residue analyses in 2020 (two samplings) and harvest. (b) Hours of sunshine, maximum and minimum relative humidity (RH), maximum and minimum temperature (T) and daily precipitation during the field trial. (c) Effect of the treatments on the mean *D. suzukii* infestation (% infested grapes ± SD). The treatments included an unsprayed control (Control), conventional spinosad treatment of the whole plant (Spinosad) and *H. uvarum* culture with spinosad treatment applied to the fruit‐free zone (H.u. + spinosad). The applied spinosad amounts were 120 g per hectare for the conventional spinosad treatment and 36.4 g per hectare for *H. uvarum* with spinosad. Treatment names followed by different lowercase letters in brackets denote significant differences in infestation between the treatments (*P* < 0.05, *n* = 3).

The concentrations and volumes used resulted in an applied amount of approximately 0.012 g of spinosad per m^2^ of treated area for the spinosad treatment and for the *H. uvarum* + spinosad treatment. The dates of application, the leaf sampling dates for the laboratory efficacy evaluation in 2019, the two leaf sampling dates and grape sampling dates for the residual analyses in 2020, and the harvest dates are in Fig. 2(a). In 2019, the second treatment was applied after an increase in *D. suzukii* infestation of grapes was observed. The harvest date was 28 September in 2019 and 23 September in 2020, which took into account the 15‐day pre‐harvest interval of the insecticide and the maturity of the grapes.

Grape samples were collected from the central row of the plot to minimize border effects. Ten samples were collected during the test period in 2019 and nine samples were collected in 2020. Each sample consisted of 50 single, blue and ripe intact berries that were cut off randomly with the berry stalk. The number of infested grape berries was counted in the laboratory with a stereomicroscope (Leica MZ 6, Leica Microsystem Srl, Milan, Italy). Eggs were visible on the surface of the grape skin by viewing the oviposition hole and two milky‐white filaments protruding out of the egg. The *D. suzukii* infestation at each timepoint was recorded as the percentage of grape berries with at least one *D. suzukii* egg. Meteorological data were obtained from the meteorological station of the Laimburg Research Centre (46°22′56.8”N, 11°17′19.5″E).

Spinosad residues were determined through liquid chromatography–tandem mass spectrometry (LC–MS/MS) as milligrams of spinosad (sum of spinosyns A and D) per kilogram of leaves or grapes during the field trial performed in 2020 following the European standard method for the analysis of pesticide residues (UNI EN 15662:2018)^31^ at the Laimburg Research Centre. One sample consisted of 17 leaves without leafstalk or approximately 300 g of grape berries. The samples were collected evenly in the central row of each block.

#### 2.3.2 Efficacy evaluation of the field application in the laboratory

Leaves from the field trial in 2019 were sampled randomly in the central row of the three plots from the treated canopy to observe the effect of the treatments on *D. suzukii* males and females in the laboratory. Samples were taken 1 and 7 days after the first application (leaf sampling 1 and 2) and 1 and 7 days after the second application (leaf sampling 3 and 4) (Fig. 2(a)). In the laboratory, five leaves from the same treatment were placed with the stalk in a 100‐mL Erlenmeyer flask filled with tap water. The Erlenmeyer flask opening was closed around the stalk with cotton and then placed in an insect cage. The cage also contained three undamaged and untreated grape berries from the control plots on a Petri dish (diameter 9 cm, polystyrene) with water agar (15 g L^–1^ agar‐agar; Merck, Darmstadt, Germany) for oviposition and cotton soaked in 10 mL of 5% sucrose solution in a small Petri dish (diameter 6 cm, polystyrene). Water agar served as an additional oviposition substrate. Twenty *D. suzukii* females and 20 males were released in the cage for 48 h. After 24 h, the berries and the water agar were replaced to count the eggs, and dead flies were removed and counted. Mortality was evaluated as the percentage of the initial number of flies, and the oviposition rate was evaluated as the number of eggs laid on the grapes and on the water agar per cage. The cages were kept under the same conditions as the *D. suzukii* rearing and arranged in a completely randomized design. Single cages were used as replicates (*n* = 6).

### 2.4 Comparison of *H. uvarum* formulations in the greenhouse

Rooted grafted vines of the variety Vernatsch (Clone: Edelvernatsch Lb 43, Rootstock: SO4) were potted in 4‐L pots filled with standard soil (SP ED63 T coarsely; Einheitserde, Sinntal‐Altengronau, Germany). The plants were grown for 2 months in the greenhouse and treated once a week for 20 min with vaporized sulfur against powdery mildew using a sulfur burner. No sulfur treatments were performed during the assay in May 2020. The mean temperature and relative humidity in the greenhouse during the assay were 21.8 °C (min. 17.3 °C, max. 29.8 °C) and 85.2% (min. 41.9%, max. 100%), respectively.

The formulations preserved in different ways by Agrifutur srl were used in this assay. Five different treatments were applied to the vines: freeze‐dried *H. uvarum* pellets dissolved in water, water + spinosad, *H. uvarum* culture + spinosad, *H. uvarum* supernatant + spinosad and freeze‐dried *H. uvarum* pellets dissolved in water + spinosad. The treatments with spinosad contained 5.43 mg of spinosad per liter of solution (11.3 μL of Laser per liter of solution), which was added after thawing and shortly before application. The chosen spinosad concentration was based on previous studies.^5^, ^8^

Each treatment was applied to 11 plants to evaluate its effect on *D. suzukii* flies and to measure the spinosad residue. Per plant, 10 leaves were marked at the stalk with a twist tie before treatment. The treatment consisted of 10 drops to 10 μL per leaf using a multichannel pipette (5–100 μL; Eppendorf Research Plus, Hamburg, Germany). One day, 7 days and 14 days after treatment, 25 leaves treated in the same way were randomly removed from the 11 plants and transferred to the laboratory.

In the laboratory, five leaves treated in the same way were immediately pooled and placed with the stalk in a 100‐mL Erlenmeyer flask filled with tap water. The opening around the stalk was closed with cotton. After that, the flask with the five leaves was placed in an insect cage. Twenty male and 20 female flies were exposed to the five leaves for 48 h. The cages also contained four nontreated blueberries (*Vaccinium corymbosum*) from organic production on a Petri dish (diameter 9 cm, polystyrene) with water agar (15 g L^–1^ agar‐agar; Merck, Darmstadt, Germany) for oviposition and cotton soaked in 10 mL of 5% sucrose solution in a small Petri dish (diameter 6 cm, polystyrene) as a water and energy source. The blueberries were washed under cool running tap water for approximately 1 min and dried with a paper towel before use. The cages were kept under the same conditions as the *D. suzukii* rearing and arranged in a completely randomized design. Single cages served as replicates (*n* = 5). After 24 h, the berries and the water agar were replaced to count the eggs, and dead flies were removed and counted. After 48 h of exposure, mortality was evaluated as a percent of the initial number of flies, and oviposition was evaluated as the number of eggs per cage.

To measure the spinosad residue amount on the leaves, one sample per treatment consisting of 10 leaves (one leaf per plant) from different positions was cut off without leafstalk 1 day, 7 days and 14 days after treatment. Samples were stored for no more than 1 month at −80 °C until analysis. The spinosad residues were analyzed by the same method as described above.

### 2.5 Statistical analyses

The *D. suzukii* infestation in the field over the entire experimental period was analyzed with a linear mixed‐effects model. The treatments were input to the model as fixed effects, while the sampling date and block were input as random effects. Tukey's pairwise comparisons were performed for the treatments.

The *D. suzukii* mortality and number of eggs laid per cage in the assays testing the efficacy of the field treatment in the laboratory and in the assays comparing the different *H. uvarum* formulations in the greenhouse were evaluated independently for each time point. Data were analyzed with a generalized linear model fitted with a gamma distribution. Datasets with zero values were *x* + 1 transformed to allow the use of a gamma distribution. The treatment and the sex of the flies entered the model as fixed effects. Models were chosen based on Akaike information criterion values, and residuals were analyzed to verify the distribution of the errors. Tukey's pairwise comparisons were performed for the treatments.

All statistical analyses were prepared with R version 4.0.2 (The R Foundation for Statistical Computing http://www.R-project.org).

## 3 RESULTS

### 3.1 Vineyard trials

#### 3.1.1 *Efficacy of the* H. uvarum *treatment in a vineyard trained with a pergola in 2019*

In 2019, the temperature and rainfall were typical for the region in August and September (Fig. 2(b)). Some rainfall was recorded after the second application and before harvest. Most days were characterized by sunshine and maximum temperatures above 25 °C.

A significant effect of the treatments on *D. suzukii* infestation over the entire experimental period occurred (*F*_2,86.82_ = 31.344, *P* < 0.001) (Fig. 2(c)). The treatment of the whole plant with spinosad and the treatment of the foliage with *H. uvarum* + spinosad reduced the *D. suzukii* field infestation significantly compared to the unsprayed control (*P* < 0.001). No differences were observed between the spinosad treatment and *H. uvarum* + spinosad treatment (*P* = 0.683).

#### 3.1.2 *Efficacy of the* H. uvarum *treatment in a vineyard trained with the Guyot method in 2020*

The experimental period in 2020 was characterized by intense rainfall over 3 days before the first application and low precipitation after the first application until harvest. Furthermore, most days were characterized by sunshine and maximum temperatures above 25 °C (Fig. 2(b)). Some *D. suzukii* infestation was already present before the first application was applied, and the *D. suzukii* infestation showed a constant increase in all treatments until harvest (Fig. 2(c)). The treatments had a significant effect on *D. suzukii* infestation (*F*_2,77.25_ = 10.9, *P* < 0.001). The foliage treatment with *H. uvarum* + spinosad significantly reduced *D. suzukii* infestation compared to the unsprayed control (*P* < 0.001). Treatment of the whole plant with spinosad also significantly reduced *D. suzukii* infestation compared to the unsprayed control (*P* = 0.003). No differences in efficacy were observed between the spinosad treatment and *H. uvarum* + spinosad treatment (*P* = 0.444).

The residue analyses showed that a lower amount of spinosad was present on the leaves treated with *H. uvarum* + spinosad, while the spinosad treatment resulted in more spinosad residue on the leaves (Table 1). In the unsprayed control treatment, no spinosad residues were found. Furthermore, no residues were detected on the untreated grapes from the control treatment and on the untreated grapes from the *H. uvarum* + spinosad treatment.

**Table 1:** Spinosad residues (mean mg/kg ± SD) on leaves and grapes sampled during the field trial and trained with the Guyot system in 2020 (*n* = 3)

	Spinosad (mg/kg)		
Treatment	Leaves Sep 04	Leaves Sep 17	Grapes Sep 17
Control	<0.01	<0.01	<0.01
Sp	1.31 ± 0.40	0.54 ± 0.20	0.05 ± 0.02
H.u. + Sp	0.16 ± 0.10	0.25 ± 0.07	<0.01

The treatments were an unsprayed control (Control), a spinosad treatment applied to the whole plant (Sp) and *H. uvarum* with spinosad treatment applied in the fruit‐free zone (H.u. + Sp).

#### 3.1.3 Efficacy evaluation of the field application in the laboratory

**Figure 3:**
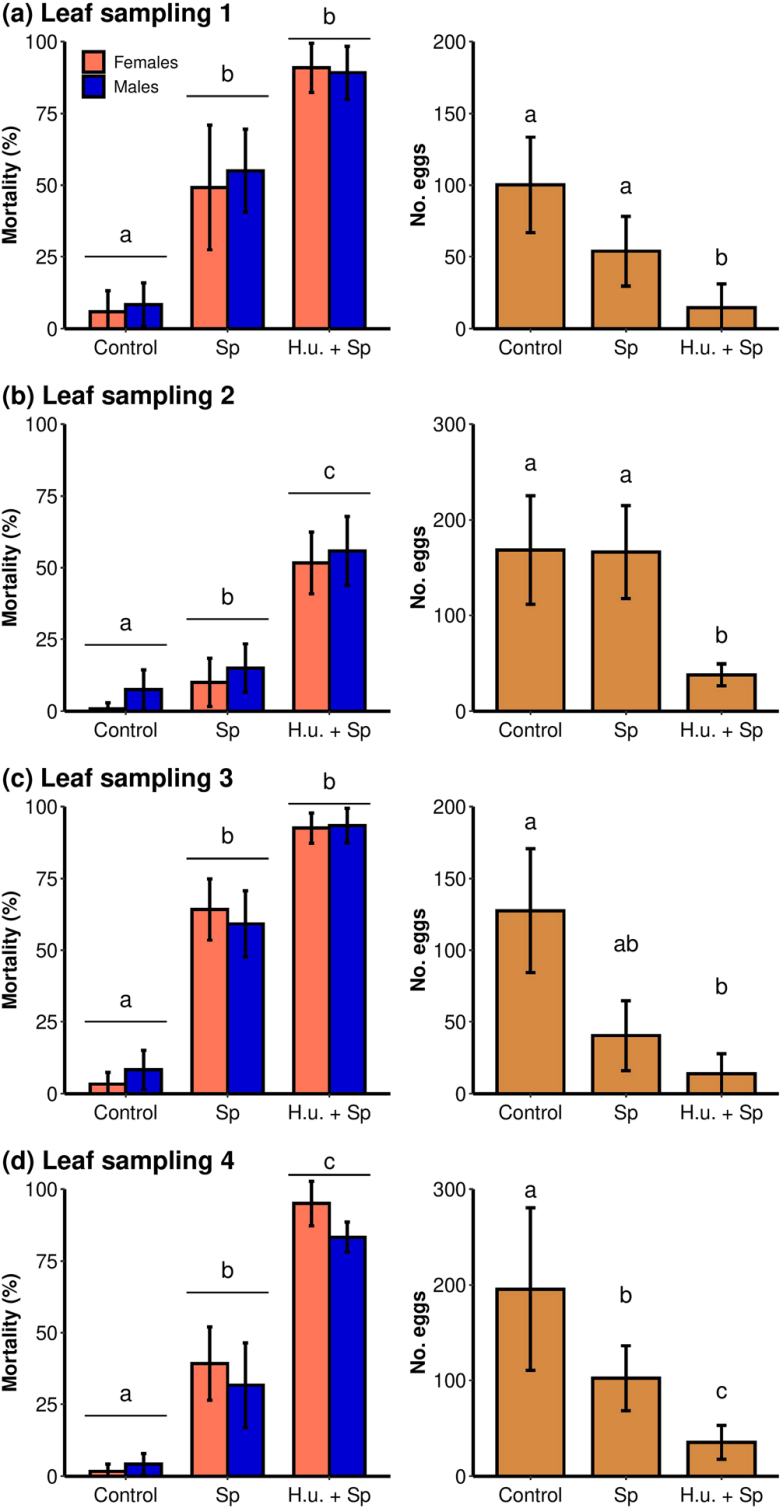
Effect of different treatments applied in the vineyard. Mean *D. suzukii* female and male mortality ± SD (left) and mean number of eggs laid per cage ± SD (right) during 48 h of exposure to leaves collected (a) 1 day and (b) 7 days after a first application and (c) 1 day and (d) 7 days after a second application. The treatments were applied in a vineyard with a pergola training system in 2019 and included unsprayed control (Control), spinosad in water (Sp) and *H. uvarum* culture with spinosad (H.u. + Sp). Different letters denote significant differences in *D. suzukii* mortality or number of eggs laid between the treatments (*P* < 0.05, *n* = 6).

No significant differences between *D. suzukii* male and female mortality were observed (leaf sampling 1: *F*_1,35_ = 0.038, *P* = 0.847; leaf sampling 2: *F*_1,35_ = 0.399, *P* = 0.532; leaf sampling 3: *F*_1,35_ = 0.001, *P* = 0.981; leaf sampling 4: *F*_1,35_ = 0.2934, *P* = 0.592). The different treatments had a significant effect on the mortality of *D. suzukii* adults (leaf sampling 1: *F*_2,35_ = 45.792, *P* < 0.001; leaf sampling 2: *F*_2,35_ = 27.228, *P* < 0.001; leaf sampling 3: *F*_2,35_ = 60.99, *P* < 0.001; leaf sampling 4: *F*_2,35_ = 74.072, *P* < 0.001) and on the number of eggs laid (leaf sampling 1: *F*_2,17_ = 9.999, *P* = 0.002; leaf sampling 2: *F*_2,17_ = 1.406, *P* < 0.001; leaf sampling 3: *F*_2,17_ = 13.733, *P* < 0.001; leaf sampling 4: *F*_2,17_ = 21.438, *P* < 0.001) (Fig. 3).

For the leaves collected 1 day after the first application (leaf sampling 1; Fig. 3(a)), both spinosad and *H. uvarum* + spinosad caused mortality over 50% and reduced the number of eggs laid by 46.7% or 83.2%, respectively. One week after application, *H. uvarum* + spinosad caused mortality of 53.8% while the spinosad treatment caused mortality of 12.5%; moreover, a significant influence on the eggs laid was not observed for spinosad without *H. uvarum* (leaf sampling 2; Fig. 3(b)). The leaves sampled after the second application (leaf sampling 3; Fig. 3(c)) confirmed the results observed 1 day after the first application. Additionally, 1 week after the second application (leaf sampling 4; Fig. 3(d)), *H. uvarum* + spinosad caused significant higher mortality and reduced oviposition compared to the control or the spinosad treatment.

### 3.2 Comparison of the *H. uvarum* formulations in the greenhouse

**Figure 4:**
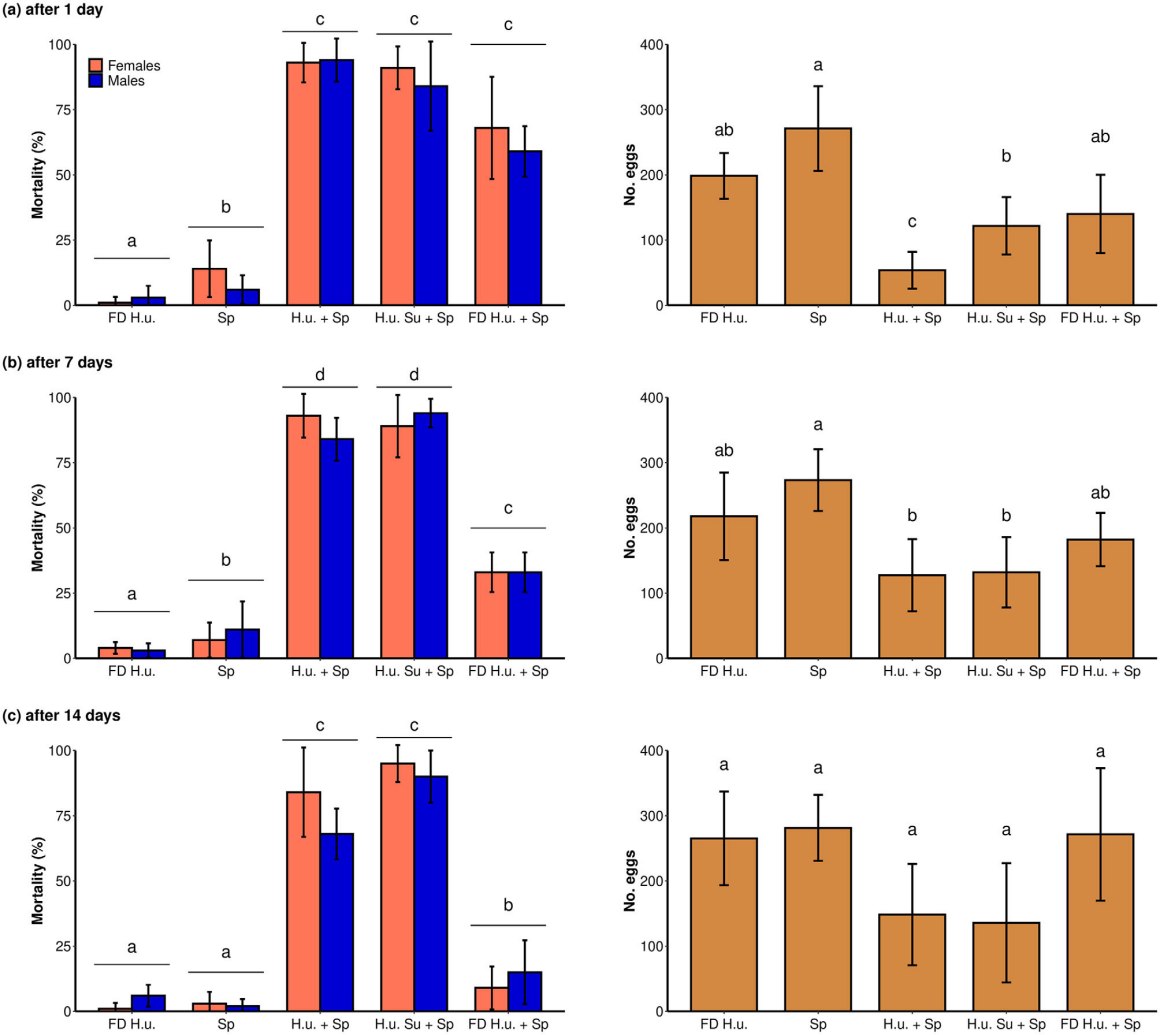
Effect of leaves treated with different *H. uvarum* formulations in the greenhouse. Mean *D. suzukii* female and male mortality ± SD (left) and mean number of eggs laid per cage ± SD (right) during 48 h of exposure to leaves collected 1 day (a), 7 days (b) or 14 days (c) after application. The treatments were applied to vine plants in the greenhouse and included an insecticide‐free formulation prepared from freeze‐dried *H. uvarum* pellets (FD H.u.), spinosad in water (Sp) and one of three *H. uvarum* formulations with spinosad. The formulations were *H. uvarum* culture + spinosad (H.u. + Sp), *H. uvarum* supernatant + spinosad (H.u. Su + Sp) and a formulation made from freeze‐dried *H. uvarum* pellets and water + spinosad (FD H.u. + Sp). Different letters denote significant differences in *D. suzukii* mortality or number of eggs laid between the treatments (*P* < 0.05, *n* = 5).

No significant differences were found in mortality between males and females (after 1 day: *F*_1,49_ = 0.121, *P* = 0.73; after 7 days: *F*_1,49_ = 0.001, *P* = 0.98; after 14 days: *F*_1,49_ = 0.039, *P* = 0.844). The different treatments had a significant effect on the mortality of *D. suzukii* adults (after 1 day: *F*_4,49_ = 35.565, *P* < 0.001; after 7 days: *F*_4,49_ = 61.14, *P* < 0.001; after 14 days: *F*_4,49_ = 38.771, *P* < 0.001) and on the number of eggs laid (after 1 day: *F*_4,24_ = 12.274, *P* < 0.001; after 7 days: *F*_4,24_ = 5.027, *P* = 0.006; after 14 days: *F*_4,24_ = 3.128, *P* = 0.038) (Fig. 4).

Over the 2‐week experimental period, all three *H. uvarum* formulations with spinosad increased mortality and reduced oviposition more than the spinosad treatment without *H. uvarum*. The spinosad treatment caused a low but significantly higher fly mortality at 1 day (10%) and 7 days (9%) after application compared to the insecticide‐free control (2%) (Fig. 4(a),(b)). Over the whole test period, *H. uvarum* culture + spinosad and *H. uvarum* supernatant + spinosad were the most effective formulations and still resulted in over 75% mortality after 2 weeks (Fig. 4(c)). In contrast, the formulation prepared with freeze‐dried *H. uvarum* pellets, water + spinosad showed a significantly lower mortality after 1 and 2 weeks compared to the other two *H. uvarum* formulations.

Analyses of the spinosad residues on the leaves from the different treatments revealed high variability (Table 2). A trend toward higher degradation of spinosad was observed in the formulation prepared of freeze‐dried *H. uvarum* pellets.

**Table 2:** Spinosad residues on leaves collected 1 day (T1), 7 days (T7) and 14 days (T14) after applying the different treatments (*n* = 1)

	Spinosad (mg/kg)		
Treatment	T1	T7	T14
FD H.u.	<0.01	<0.01	<0.01
Sp	NA^a^	0.41	0.34
H.u. + Sp	1.29	0.78	0.35
H.u. Su + Sp	0.62	0.36	0.29
FD H.u. + Sp	0.64	0.13	0.06

^a^ Not available due to a measurement error.

The treatments were a formulation made from freeze‐dried *H. uvarum* pellets and water (FD H.u.), water + spinosad (Sp), *H. uvarum* culture + spinosad (H.u. + Sp), *H. uvarum* supernatant + spinosad (H.u. Su + Sp) and the formulation made of freeze‐dried *H. uvarum* pellets and water + spinosad (FD H.u. + Sp).

## 4 DISCUSSION

In field trials over 2 years, yeast cultures of *H. uvarum* with spinosad were compared with spinosad applications to control *D. suzukii* in vineyards. The results showed that targeted treatment of the foliage without spraying the berries of the grapevines was possible for both the pergola and Guyot methods, and as efficient pest control as the conventional insecticide treatment of the whole plant. Compared to the application of spinosad on the whole plant, the treatment based on *H. uvarum* with the addition of spinosad that was applied only on the foliage did not leave spinosad residue on the fruits at harvest. Furthermore, the amount of spinosad applied in the *H. uvarum* treatment was 36.48 g of spinosad per hectare, which was approximately three times lower than that in the conventional treatment with 120 g of spinosad per hectare. Based on these results, the proposed pest control strategy based on *H. uvarum* and spinosad could be a practicable alternative to typical insecticide applications.

*H. uvarum* bait with spinosad targets highly mobile adults. Control of adult *D. suzukii* is important because flies immigrating into vineyards from noncrop hosts cause initial infestations.^32^, ^33^, ^34^ Since *D. suzukii* females are more attracted to the fruit than to the grapevine leaves, a promising strategy is the application of the bait evenly on the foliage, thus creating a multitude of attractive points to reduce the attraction to the fruit and increase the attraction to *H. uvarum*‐treated leaves.^19^, ^35^ After attraction, the flies readily come into contact with the insecticide as they stay on the leaves to feed. Another aspect is the persistence of the attractiveness of the bait and the insecticidal effect of the insecticide. The evaluation of the leaves in the laboratory showed that the *H. uvarum* bait was still effective after 1 week and up to 2 weeks with two applications. Since spinosad loses some effect after 1 week,^22^ the treatments should be applied at intervals of 1 week to 2 weeks based on the *D. suzukii* infestation, predicted rainfall and precipitation quantity after application, which could affect the efficacy by washing off the *H. uvarum* bait and the insecticide. The results from the laboratory showed the higher efficacy of spinosad in the *H. uvarum* treatment and confirmed that *H. uvarum*‐treated leaves were indeed more effective.^18^ Since the applied dose of active ingredient per area treated zone was not reduced in the *H. uvarum* treatment, no negative effects on resistance development are to be expected. As different insecticides can be used in combination with *H. uvarum*,^18^, ^19^ exchanging the active ingredients could also reduce the risk of developing resistance, such as to spinosad,^36^, ^37^ and allow for the application of this control strategy to crops for which spinosad is not registered.

For practical uses in agriculture, an issue could be the difficulty in obtaining a stable *H. uvarum*‐based formulation.^19^, ^20^ For commercial use, a stable and dry product would simplify marketing and use by farmers. Freeze‐dried *H. uvarum* pellets were tested since smaller volumes reduce the costs of storage and transport. A second possibility would be the elimination of *H. uvarum* cells. The storage of a sterile product without living cells does not require any special preservation to maintain vitality, and the cells can deposit in the sprayer. In this study, the supernatant without *H. uvarum* cells had a similar efficacy as the whole *H. uvarum* culture (both stored at −80 °C before application) and both retained their effect over 2 weeks. This finding was not surprising since the largest part of the yeast metabolites in a similarly grown culture of *H. uvarum* was in the supernatant and not in the yeast cells.^10^ The freeze‐dried *H. uvarum* pellets dissolved in water lost some efficacy. Although a prior centrifugation step reduced the effort and energy requirements compared to freeze‐drying the entire *H. uvarum* culture, it also probably caused the loss of important components in the supernatant. Furthermore, leaves treated with freeze‐dried *H. uvarum* pellets dissolved in water with spinosad showed the lowest spinosad residues after 14 days. Numerous factors affect the stability of spinosad, such as photolysis and biotic degradation.^38^ Further studies are necessary to determine the reasons for the differences in spinosad residues observed under the experimental conditions reported in this study.

In addition to the necessary improvement of the formulation, new emerging precision technologies could simplify the implementation of the proposed targeted treatment strategy for fruit‐free canopies and reduce the drift of the yeast formulation onto weeds and surrounding vegetation, and thus the hazards to nontarget organisms.^39^ On the pergola, the treatment can be applied by one application from below to the canopy, while with the Guyot system, the treatment can be applied from both sides to the canopy. On the Guyot system, the grapes are below the treated canopy, therefore the probability of dripping from the treated canopy to the grapes is higher. No spinosad residues were found on the grapes, therefore it can be assumed that at an application rate of 230 L of yeast culture per hectare, no notable dripping to the fruit occurred. In the pergola system, the canopy‐ and grape‐containing zones are not on top of each other, which reduces the risk of dripping on the grapes. Therefore, residues on the grapes are less likely. For small‐scale field applications, the spraying of *H. uvarum* with spinosad bait using a knapsack sprayer can provide an alternative for the control of *D. suzukii*. Advantages result from the applicability in different training systems and manual application, which allows for the easy and selective treatment of the canopy. Further improvements should focus on the development of spraying equipment for large‐scale vineyards, which allows for fast and precise application due to the automatic limitation of the application to the grape‐free canopy.

## 5 CONCLUSION

The yeast *H. uvarum* can be used for attract‐and‐kill control strategies against *D. suzukii* under the conditions proposed in this study. The advantages of this method in terms of sustainable control measures are associated with the lower amount of residual spinosad on the fruits and the reduced amount of insecticide applied in the environment. Commercial and storable formulations based on *H. uvarum* should avoid the loss of the supernatant, which contains attractive and feeding stimulant compounds, while the preservation of living yeast cells seems to be less important. Further studies are needed to explore the efficacy of this technique on other fruit crops and to develop a stable, easy‐to‐store and ready‐to‐use product.

## CONFLICT OF INTEREST

The authors declare that they have no conflicts of interest.

## Data Availability

The data that support the findings of this study are available from the corresponding author on reasonable request.
